# Small bowel masses

**DOI:** 10.1186/1470-7330-15-S1-O10

**Published:** 2015-10-02

**Authors:** Gabriele Masselli

**Affiliations:** 1 Policlinico Umberto I University Sapienza, Rome, Italy

## 

The diagnosis of small-bowel tumours, particularly early detection and differential diagnosis, is still somewhat challenging, although many sensitive direct and indirect techniques have been adopted.

Although spatial resolution of MR imaging is lower than that of a CT scan, the main advantages of the former are the combination of good soft-tissue contrast, detection of extraenteric abnormalities, and lack of radiation exposure, which allows repeated data acquisition for functional bowel evaluation.

Enteroclysis provides greater distention of the entire small bowel than does enterography in patients suspected of having small-bowel neoplasms, because small polypoid masses that do not produce obstruction are most likely difficult to detect by using oral contrast material distention.

Moreover, MR enteroclysis delineates superficial changes more accurately than does MR enterography and the evaluation of endoluminal abnormalities is particularly important in the detection of early-stage small-bowel neoplasms.

MR enteroclysis has yielded a 96.6% accuracy in the detection of small-bowel neoplasms, thereby proving to be an effective means of diagnosing or ruling out small-bowel neoplasms.

MR enterography is an accurate, well-tolerated, promising imaging modality with which to diagnose or exclude small-bowel tumours in symptomatic patients with negative upper and lower endoscopy findings. MR enterography might allow clinicians to select patients in whom more invasive diagnostic methods are indicated.

MR enterography, which allows improved localization of small-bowel polyps in patients with Peutz- Jeghers syndrome, is performed to identify larger lesions that should be resected at double-balloon enteroscopy or surgery. It may also be helpful for excluding the presence of lesions in bowel segments not examined at endoscopy or surgery.

MRI has proved to be more sensitive than CT for the detection of endoluminal esions of the small bowel, owing to improved detection of segments with subtle abnormalities.

These findings may be due both to the better soft-tissue contrast afforded by MR imaging, which is required for tissue characterization and the detection of subtle areas of abnormality, and to its functional capabilities. Another advantage of MR imaging over CT is that the enhanced soft-tissue contrast produced by MR imaging may provide more information regarding the nature of mesenteric small bowel tumors, thereby allowing better characterization of small-bowel tumors. In this regard, benign tumors such as hemangiomas are typically strongly hyperintense on T2-weighted MR images, whereas lipomas or tumors with a marked fat content are spontaneously hyperintense on T1-weighted MR images.

With few exceptions (lymphoma being one), thickening of a long segment of the small bowel is indicative of a benign condition.

MR enteroclysis and enterography are accurate non-invasive modalities in assessing the intraluminal, parietal and extraluminal neoplastic manifestations. MR signal appearances of the lesions, combined with the contrast enhancement behaviour and the characteristic of the stenosis, can help in differentiating from other non-neoplastic diseases of the small-bowel.

Radiologists should therefore be familiar with the MR appearance of various small-bowel neoplasms and their mimickers.
